# Prolonged water-only fasting in the management of low-grade follicular lymphoma: a case series

**DOI:** 10.1186/s13256-024-04609-w

**Published:** 2024-07-03

**Authors:** Sahmla Gabriel, Toshia R. Myers, Natasha Thompson, Alan C. Goldhamer

**Affiliations:** 1 TrueNorth Health Foundation, Santa Rosa, CA USA; 2 TrueNorth Health Center, Santa Rosa, CA USA

**Keywords:** Follicular lymphoma, Prolonged water-only fasting, Whole-plant-food diet, Alternative therapy, Nutritional therapy, Case report

## Abstract

**Background:**

Follicular lymphoma typically follows an indolent and relapsing course often requiring several treatment cycles to achieve remission. Some patients opt to use complementary and alternative therapies particularly when observation is a treatment option.

**Case presentation:**

Here we present a case series of three patients, a 50-year-old, White, Hispanic female, 56-year-old, White, non-Hispanic male, and 49-year-old, White, non-Hispanic male, who elected to undergo one or more prolonged water-only fasting and refeeding interventions to manage low to intermediate grade follicular lymphoma. Fasting was well tolerated in each patient. Each patient also experienced a reduction in the size and avidity of hypermetabolic lymph nodes as independently determined by their respective oncologists.

**Conclusion:**

The reported cases demonstrate positive outcomes in low-grade follicular lymphoma coinciding with prolonged water-only fasting and exclusively whole-plant-food dietary interventions. These findings highlight the potential of such interventions and warrant further exploration through preliminary observational research.

## Background

Follicular lymphoma (FL) is a prevalent form of non-Hodgkin lymphoma characterized by the gradual expansion of abnormal B cells, known as lymphoma cells, within follicles, or nodules, that can accumulate in the lymphatic system and various tissues throughout the body. FL development is heterogenous and evaluated based on histological grade (1, 2, 3a, 3b) and stage (I, II, III, IV, bulky), determined through imaging and clinical presentation. Treatment strategies range from observation or localized radiation for non-aggressive, slow-growing, low-grade FL to more intensive approaches such as chemotherapy, immunotherapy, radiation, or combination therapy for aggressive, high-grade, and/or advanced stage FL. Improvements in immunotherapy have increased five-year survival rates, but patients may still need to undergo multiple treatments to achieve FL remission. Additionally, relapses commonly occur within 1 to 2 years after the initial remission [1].

Fasting is emerging as a promising tool in cancer therapy as evidenced by research suggesting that various periods of controlled nutrient deprivation may enhance the effectiveness of established cancer treatments, mitigate treatment-related side effects, and impede tumor growth [2–4]. Indeed, a growing number of cancer patients are opting to manage the disease with fasting and other complementary and alternative therapies while maintaining diagnostic care with an oncologist [5]. Prolonged water-only fasting is an established and safe method of therapeutic fasting during which patients consume only water for up to 40 days followed by gradual refeeding under medical supervision [6]. Previously, we reported on a female patient with stage IIIa, low-grade FL who achieved sustained remission that coincided with a 21-day water-only fast [7]. Here, we present a 10-year follow-up report of that case as well as the cases of two other patients with a diagnosis of low-grade FL who opted to undergo prolonged water-only fasting in order to manage the disease.

## Case presentation

The patients presented here received a diagnosis of FL by their respective oncologists, and each patient independently opted to manage the cancer with prolonged water-only fasting and refeeding while at a residential fasting center receiving medical supervision and according to an established protocol [6]. Briefly, the patients were approved for fasting after thorough medical evaluation, which included clinical, serological, and urinalysis to monitor for potential contraindications [6]. They prepared to fast by eating a diet of fresh fruits and fresh and steamed vegetables ad libitum for two days. Once fasting commenced, they were instructed to consume a minimum of 1.2 L of distilled water per day and reduce daily physical activity. The patients’ vitals were monitored twice daily by medical personnel, and serology (i.e., complete blood count, comprehensive metabolic panel, and additional testing as medically indicated) and basic urinalyses were measured once weekly and as medically indicated. Each patient’s fast was terminated with the gradual reintroduction of food according to the standard refeeding protocol [6]. Briefly, food reintroduction occurred over a duration of at least half of the fast length and consisted of 5 stages beginning with fresh fruit and vegetable juices followed by the gradual introduction of whole-plant foods, with each phase increasing in complexity until patients were eating a full-range of whole-plant foods free of added salt, oil, or sugar (SOS-free diet) [6].

## Case 1

We previously published the case of a patient with stage IIIa, low-grade FL who achieved complete remission for three years after a single intervention of prolonged water-only fasting followed by an SOS-free diet [7, 8]. Nine years after the initial intervention, the 50-year-old, White, Hispanic female returned to our residential fasting center to undergo a third prolonged water-only fast with the intention of supporting continued FL remission and reducing weight gained after having contracted SARS-CoV-2. The patient reported that she had maintained yearly follow-up oncology appointments that included serological and computed tomography (CT)/positron-emission tomography (PET) examinations, which confirmed her continued remission.

**Fig. 1:**
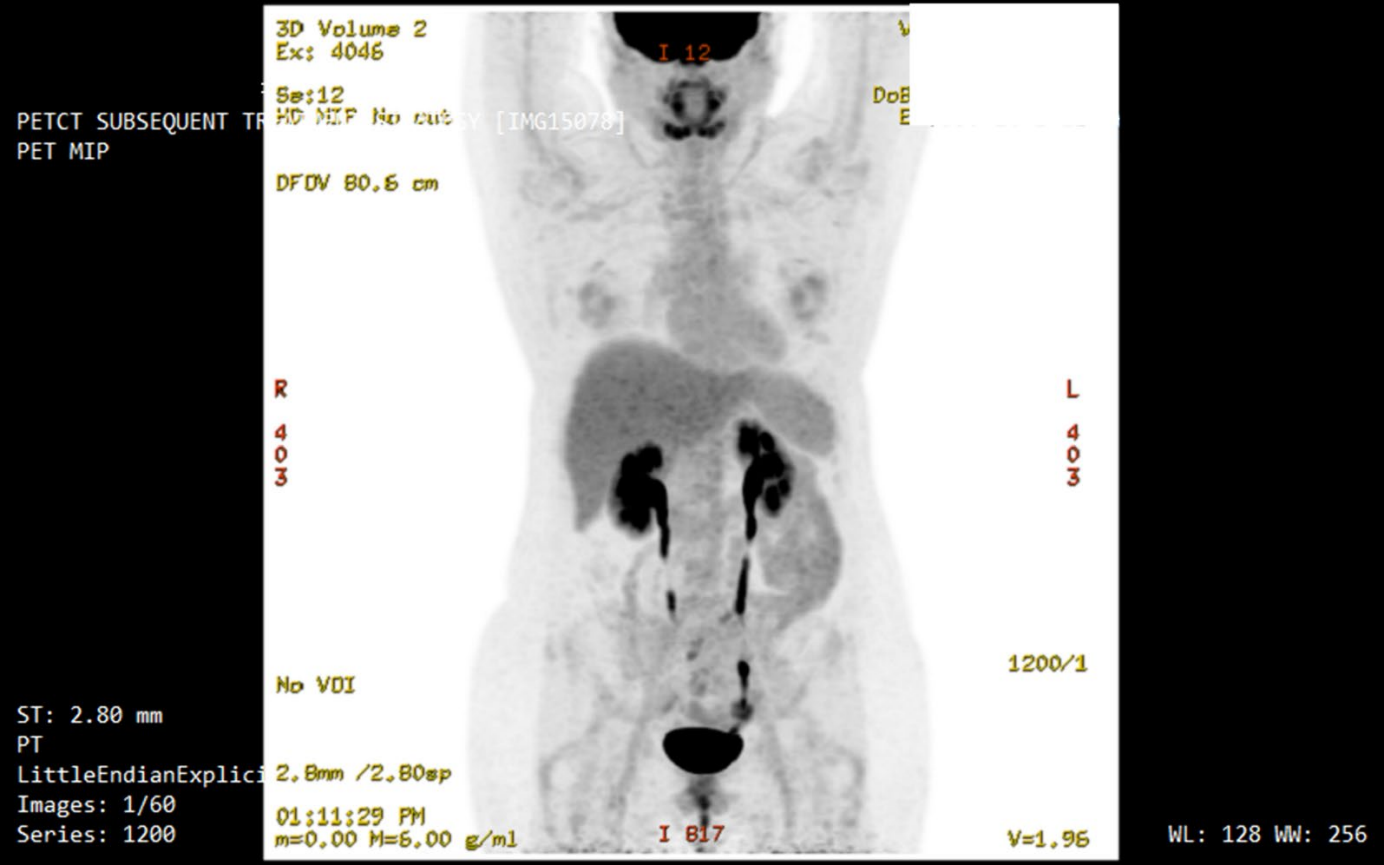
Maximum intensity projection (MIP) positron emission tomography PET image after patient’s third fast occurring 10 years after her initial diagnosis

On arrival, the patient had no significant health complaints, was not taking any medications, and had unremarkable clinical and serological exams. She weighed 72.4 kg with a body mass index (BMI) of 27.3 kg/m^2^ and systolic/diastolic blood pressure of 106/72 mmHg (Table 1). She completed 18 days of fasting, during which she experienced mild nausea and dry mouth, and 10 days of refeeding that was well tolerated. On the 12th day of fasting, standard serology indicated that she had developed mild hypokalemia with a serum potassium level of 2.9 mmol/L. Other electrolytes tested, including sodium, remained within normal limits, and her neurologic and cardiologic clinical evaluations were unremarkable. She consumed 350 mL of low-calorie vegetable broth four times per day for the next six days until refeeding began. By the end of refeeding, she weighed 67.3 kg with a BMI of 25.4 kg/m^2^ and systolic/diastolic blood pressure of 102/69 mmHg (Table 1). On discharge, the patient was in good health. Her yearly oncological evaluation was completed ten months later and revealed serology within normal limits and a MIP PET scan with no evidence of malignancy in the chest, abdomen, and pelvis (Fig. 1).

**Table 1:** Body weight, body mass index, and blood pressure before, during, and after water-only fasting

Case (#)	Intervention (d, phase)	BW (kg)	BMI (kg/m^2^)	BP (mmHg)
1	0, P_3_	72.4	27.3	106/72
	18, F_3_	65.7	24.8	88/58
	26, R_3_	67.3	25.4	102/69
2	0, P_1_	71.5	22.0	109/70
	22, F_1_	61.3	18.8	113/82
	29, R_1_	61.7	19.0	99/67
	0, P_2_	79.4	24.4	131/67
	40, F_2_	64.4	22.7	119/73
	54, R_2_	65.8	20.2	108/71
	0, P_3_	82.4	25.3	152/81
	40, F_3_	67.6	20.8	112/72
	55, R_3_	70.8	21.8	104/72
3	0, P_1_	82.6	27.5	120/86
	21, F_1_	69.9	23.2	107/70
	31, R_1_	72.9	24.3	99/65

d, day; kg, kilogram; BW, body weight; BMI, body mass index; m, meter; BP, blood pressure; mmHg, millimeter of mercury; P, prefeeding; F, fasting; R, refeeding; _1_, first fast; _2,_ second fast; _3_, third fast

## Case 2

**Fig. 2:**
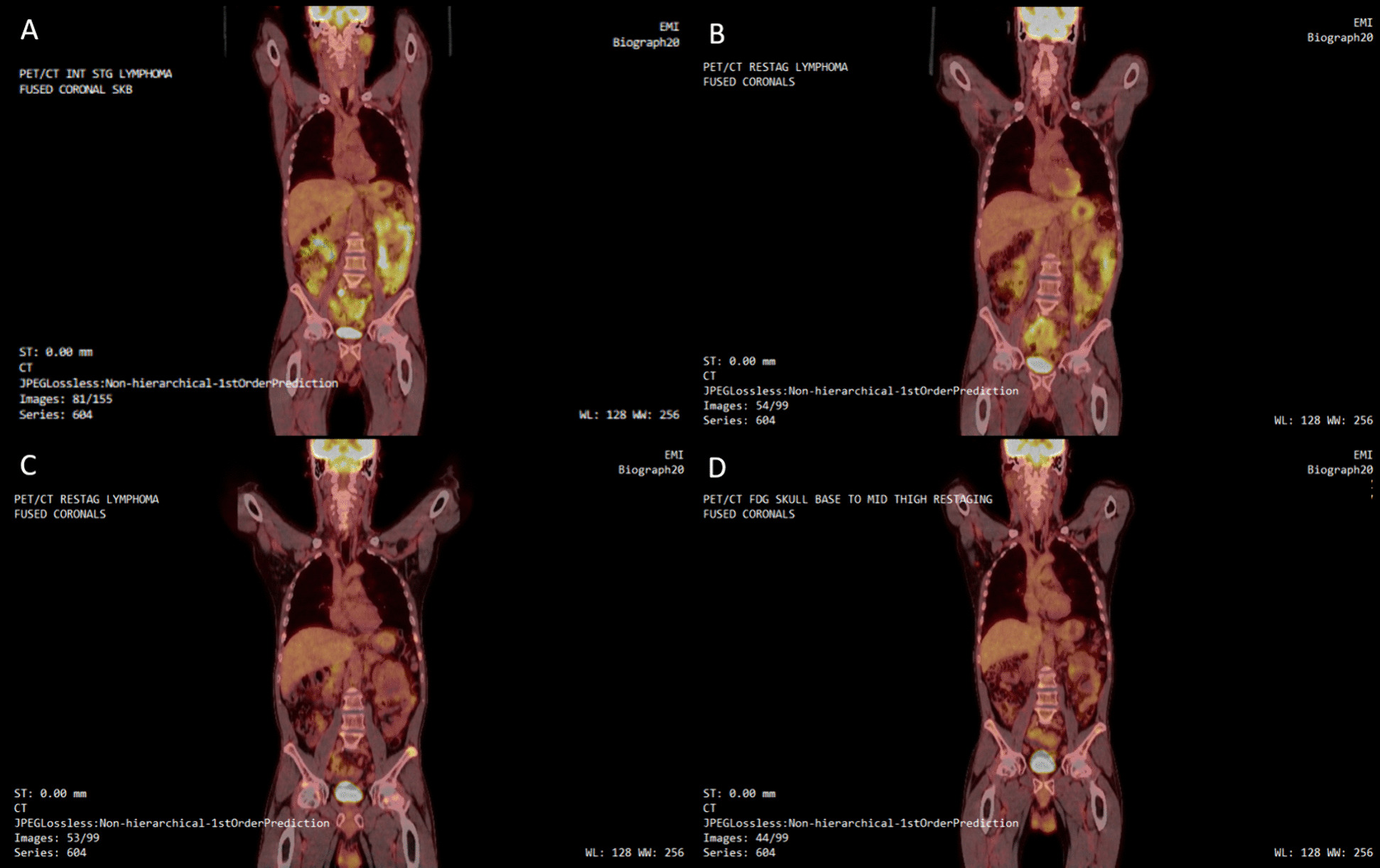
F-18 fluorodeoxyglucose (^18^F-FDG) positron emission tomography (PET) and/or computed tomography (CT) images: **A** before fasting; **B** two months after first fast; **C** five months after second fast; **D** nine months after second fast occurring over a span of two years

A 56-year-old, White, non-Hispanic male arrived to the residential fasting center with a non-tender palpable mass in his right inguinal region that was diagnosed as stage III, grade 2 FL six months earlier based on findings from a CT/PET scan and biopsy. The diagnostic scan revealed hypermetabolic mesenteric and retroperitoneal lymphadenopathy with significant lymph node size and avidity (Table 2). See Fig. 2A for whole-body ^18^F-FDG PET/CT scan upon original diagnosis. The oncologist recommended chemotherapy, but the patient opted to begin treatment with a prolonged water-only fast. On arrival, he weighed 71.5 kg with a BMI of 22.0 kg/m^2^ and systolic/diastolic blood pressure of 109/70 mmHg (Table 1). He reported that he did not take any prescription medications and that he quit smoking the previous year after 35-years of tobacco use, consumed alcohol daily, and used marijuana on weekends. He also had a history of diverticulosis that often led to abdominal pain and bloating but reported milder symptoms since switching to a plant-food diet eight years earlier, which also resulted in a weight loss of 23 kg.

**Table 2:** Case 2 F-18 fluorodeoxyglucose positron emission tomography and/or computed tomography Analysis Before and After Water-only Fasting

	Lymph node type/size (cm)/FDG avidity (SUVmax)*			
	Diagnostic scan	2 months after First Fast	3 months after second fast	11 months after second fast
Head/neck	No abnormal findings	No abnormal findings	No abnormal findings	No abnormal findings
Chest	No abnormal findings	No abnormal findings	No abnormal findings	• Right axillary/0.7 /2.1
Abdomen/Pelvis	• Mesenteric/ 3.2 × 4.5/ 6.8 • Abdominal/2.0 × 2.8 / 5.3 • Periaortic/1.2 × 2.2 / 2.7 • Left femoral/1.5 × 2.1 / 7.8	• Mesenteric/2.2 × 4.0/ 3.5 • Abdominal/”Prominent”/ < 2.0 • Left femoral/0.5 × 1.3/ < 2.0	• Left upper quadrant mesenteric/ 2.4 × 1.6/ 5.6	• Mesenteric/”Stable prominence”/ 4.1 • Right external iliac/0.8/ 4.4
Osseous	No abnormal findings	No abnormal findings	• Left iliac bone/NA/5.2 • Right iliac bone/NA/3.8 • Left rib/NA/2.8 • Thoracic spine/NA/ 3.2	• Focal uptake at T11/NA/4.9 • Focal uptake at L3/NA/ 2.9
Impressions	▪ Mesenteric and retroperitoneal lymphadenopathy ▪ Consistent with diagnosis lymphoma	▪ Mesenteric and retroperitoneal lymphadenopathy decreased in size since prior exam ▪ Abdominal lymphadenopathy decreased in size and avidity ▪ Consistent with partial treatment^^^ response	▪ Interval decrease in prominence of multiple mesenteric lymph nodes with stable to minimal increase in uptake ▪ Diffuse multifocal osseous uptake ▪ Lymphomatous involvement of osseous structures is indeterminate	▪ Small right external iliac and right axillary lymph nodes demonstrate uptake ▪ Diffuse multifocal osseous uptake, slightly decreased compared to prior analysis ▪ Lymphomatous involvement of osseous structures is indeterminate

^*^Independent scans are not necessarily comparing the same lymph nodes. ^^^ Treatment consisted only of water-only fasting. FDG, fludeoxyglucose F18; SUVmax, maximum standardized uptake value; cm, centimeter; NA, measurement not applicable

The patient was thoroughly examined and approved to water-only fast. He completed a 21-day fast with mild and moderate symptoms including decreased energy levels, decreased sleep quality and duration, mild abdominal pain, and mild acid reflux in the early days of fasting. All of these symptoms subsided within nine days of refeeding. By the end of refeeding, the main right inguinal mass was no longer palpable; he weighed 61.7 kg with a BMI of 19.0 kg/m^2^ and systolic/diastolic blood pressure of 99/67 mmHg (Table 1). Two months later, he underwent another CT/PET scan, which revealed a reduction in the size and fludeoxyglucose F18 (FDG) avidity of abdominal and pelvic lymph nodes (Table 2). See Fig. 2B for whole-body ^18^F-FDG PET/CT scan after the first fast. Based on these finding as well as normal serology, the oncologist advised for observation and to repeat diagnostic scans in six months.

The patient remained under the care of an oncologist, maintained a whole-plant-food diet, and made efforts to decrease alcohol and marijuana use. Ten months after the first intervention, the patient returned to the residential fasting center and completed a second water-only fast of 39 days without serious adverse events. At the beginning of the second fast, he weighed 79.4 kg with a BMI of 24.4 kg/m^2^ and systolic/diastolic blood pressure of 131/67 mmHg (Table 1). At the end of the 15-day refeeding period, he weighed 65.8 kg with a BMI of 20.2 kg/m^2^ and systolic/diastolic blood pressure of 108/71 mmHg (Table 1). Five months after the second fast, the patient underwent his yearly CT/PET scan, which revealed that mesenteric lymph nodes had reduced in size and that inguinal nodes were undetectable. See Fig. 2C for whole-body ^18^F-FDG PET/CT scan five months after second fast. The scan also revealed a new finding of multifocal osseous uptake in the left iliac with a maximum standardized uptake value (SUVmax) of 5.2, right iliac with a SUVmax of 3.8, left posterior rib with a SUVmax of 2.8, and lower thoracic spine with a SUVmax of 3.2, which was found to be “indeterminate” for lymphomatous involvement (Table 2). Based on the nodal response and the possibility of acute reactive changes on imaging combined with normal clinical and serological examinations the oncologist recommended deferring additional biopsy and having a shorter-term follow-up visit [9]. The exam was repeated nine months later and revealed stable mesenteric findings as well as decreased osseous uptake (Table 2). See Fig. 2D for whole-body ^18^F-FDG PET/CT images nine months after second fast.

Two years after second fast, the patient returned to the residential fasting center with the intention of completing another prolonged water-only fast. He weighed 82.4 kg with a BMI of 25.3 was kg/m^2^ and systolic/diastolic blood pressure of 152/81 mmHg (Table 1). He completed a 40-day water-only fast without any severe or serious adverse events. Throughout the fast, he experienced mild to moderate decreased energy levels, decreased sleep quality and duration, and nausea that was relieved with carbonated water. At the end of the 17-day refeeding period, he weighed 70.8 kg with a BMI of 21.8 kg/m^2^ and systolic/diastolic blood pressure of 104/72 mmHg (Table 1). He no longer had any palpable masses and reported that he will continue with yearly follow-up appointments with the oncologist where he is monitored via serology and CT/PET scan.

## Case 3

A 49-year-old, White, non-Hispanic male arrived at the residential fasting center with the intention of undertaking a prolonged water-only fast to manage the recurrence of FL. Two years prior, he was diagnosed with grade 3a FL of the head and neck and achieved remission with radiation therapy. One year later, a surveillance CT/PET scan identified several small hypermetabolic mesenteric lymph nodes, the most prominent measuring 1.9 cm × 1.5 cm with a with SUVmax of 2.9, 1.9 cm × 1.2 cm with a with SUVmax of 5.0, and 1.2 cm × 0.8 cm with a with SUVmax of 3.6 (Table 3). He also had a history of benign prostatic hyperplasia with nocturia. He did not take any medications, did not smoke nor consume alcohol, and ate a whole-plant-food diet. On arrival, he weighed 82.6 kg with a BMI of 27.5 kg/m^2^ and systolic/diastolic blood pressure of 120/86 mmHg (Table 1). He was thoroughly examined and approved to water-only fast. The patient fasted for 21 days during which he experienced mild fatigue and sleep disturbances that subsided upon refeeding. At the end of the 11-day refeed, he weighed 72.9 kg with a BMI of 24.3 kg/m^2^ and systolic/diastolic blood pressure of 99/65 mmHg (Table 1). On discharge, he was instructed to resume care with the oncologist.

**Table 3:** Case 3 F-18 fluorodeoxyglucose positron emission tomography and/or computed tomography Analysis Before and After Water-only Fasting

	Lymph node type/size (cm)/FDG Avidity (SUVmax)	
	Surveillance scan^*^	2 months after fast
Head/Neck	No abnormal findings	No abnormal findings
Chest	No abnormal findings	Axillary/”Small”/”Low-level avidity”
Abdomen/ Pelvis	• Mesenteric/ 1.9 × 1.5/ 2.9 • Mesenteric/ 1.9 × 1.2/ 5.0 • Mesenteric/ 1.2 × 0.8/ 3.6	• Mesenteric /1.7 × 0.7/ 3.6 • Mesenteric/ 2.6 × 0.9/ 3.4 • Inguinal/”Normal”/“Low-level avidity”
Osseous	No abnormal findings	No abnormal findings
Impression	▪ No residual abnormality at the level of the head or neck ▪ No visceral manifestations of malignancy	▪ Improvement in size and metabolic activity of mesenteric lymph nodes ▪ No evidence of hypermetabolic malignancy or new disease

^*^Scan after 1 year of remission achieved through radiation therapy. FDG, fludeoxyglucose F18; SUVmax, maximum standardized uptake value; cm, centimeter

At a follow-up visit, the patient reported that he continued eating a whole-plant-food diet and that three months after the intervention, a CT/PET scan indicated that the size and avidity of his mesenteric lymph nodes had improved. Of note, there were only two prominent nodes in comparison to three in the previous scans, now measuring 1.7 cm × 0.7 cm with a SUVmax of 3.6 and 2.6 cm × 0.9 cm with a SUVmax of 3.4, and there was no other evidence of hypermetabolic malignancy in any other compartments (Table 3). His oncologist advised for observation with yearly follow-up visits.

## Discussion

The cases presented in this series suggest there may be a potential use for prolonged water-only fasting followed by a whole-plant food diet as a low-risk, adjunctive therapy to manage low-grade FL, particularly during periods of watchful waiting. These three patients underwent one or more prolonged water-only fasting and whole-plant-food refeeding interventions as an alternative treatment for low-grade FL. Two of the patients did not have conventional cancer treatments and engaged in multiple fasting and refeeding periods over several years as part of their long-term management strategy. The third patient initially achieved remission of FL with radiation, but when the cancer relapsed one year later, he opted to manage the disease beginning with a single fasting and refeeding intervention. These interventions coincided with demonstrable and sustained positive outcomes in the size and avidity of hypermetabolic lymph nodes. The patients all reported eating a whole-plant-food diet before and after the fasting intervention, which may have contributed to outcomes through various potential mechanisms but appears to have been insufficient to initiate remission in these cases. Although there are also demographic, lifestyle, and environmental differences between these cases that may have affected individual outcomes, at the time of publication, all patients were watchful waiting under the observational care of their oncologist.

Prolonged water-only fasting leads to sustained reductions in weight, fatty liver index, and markers of inflammation [10], all of which might enhance immunity or affect other physiological functions that could potentially improve the body's natural ability to eliminate cancer. Animal research suggests several mechanisms by which fasting may regulate tumor growth including metabolic changes, increased autophagy, enhanced immunity, and decreased angiogenesis, but these potential mechanisms have not been validated in humans [11, 12]. Human research into the effects of fasting as an adjunctive therapy in patients suggests short water-only fasts before and after conventional treatments may improve efficacy and reduce treatment-induced adverse events [13, 14]. The effects of longer period of water-only fasting in this context have not been investigated. Despite inherent limitations to clinical anecdotes, the outcomes observed in this case series set a precedent for preliminary research supporting the clinical trials needed to assess the causality and efficacy of prolonged water-only fasting and whole-plant-food diet in the treatment of low-grade FL.

## Conclusion

The reductions in the size and avidity of hypermetabolic lymph nodes presented in this report suggest that prolonged water-only fasting followed by whole-plant-food refeeding may be a potential low-risk option for managing FL. These clinically meaningful outcomes support further inquiry into the safety, feasibility, and efficacy of prolonged water-only fasting in the management of FL, particularly during observational periods when the patient is waiting to see if the disease will progress or for patients opting out of conventional treatment.

## Data Availability

Data sharing not applicable to this article.
